# Evaluation of clinical factors influencing pregnancy rate in frozen embryo transfer 

**Published:** 2014-07

**Authors:** Maryam Eftekhar, Elham Rahmani, Soheila Pourmasumi

**Affiliations:** 1 *Research and Clinical Center for Infertility, Shahid Sadoughi University of Medical Sciences, Yazd, Iran.*; 2 *Department of Obstetrics and Gynecology, Bushehr University of Medical Science, Bushehr, Iran.*

**Keywords:** *Frozen embryo transfer*, *In Vitro Fertilization*, *Clinical pregnancy rate*

## Abstract

**Background:** Frozen embryo transfer (FET) is one of the most important supplementary procedures in the treatment of infertile couples. While general information concerning the outcome of fresh embryo transfer has been documented, paucity of investigations has addressed the clinical factors influenced on pregnancy rates in FET.

**Objective:** In this study, we performed a retrospective analysis of clinical factors that potentially influence the outcome of FET.

**Materials and Methods: **We reviewed the data from 372 women who were subjected to FET registered from April 2009-2011 at the Research and clinical center, Shahid Sadoughi University of Medical Sciences, Yazd, Iran. Baseline data and pregnancy rate were collected. The data were analyzed statistically using the Kolmogorov-Smirnov, and Mann-Whitney tests.

**Results: **The clinical pregnancy rate was 57.7 and 29.2% in women <35 years old, and women >35 years old, respectively (p<0.0001). Clinical pregnancy rates in women with FSH <10 IU/ml, and FSH >10 IU/ml were 56.3% and 17.5 %, respectively (p<0.0001). Whereas the other clinical parameters consist of reason of fetus freezing, primary IVF protocol, IVF procedure, endometrial thickness, treatment duration to fetal transfer found to be unrelated to FET outcomes (p>0.05).

**Conclusion:** Female age and basal FSH level are the most important factors influencing the clinical pregnancy rate following FET.

## Introduction

Since the first successful reports of frozen embryo transfer (FET), the cryopreservation of embryos has been an important supplementary procedure in the treatment of infertile couples (1-2). In the recent years, FET has become an important component of assisted reproductive technology (ART) (3). This procedure provides the means to reduce the number of transferred embryos, as well as, contributes to lowering the risk of multiple pregnancies (4-5). On the other hand, embryo cryopreservation has also provided additional clinical safety in the presence of ovarian hyperstimulation (5-10). However, the pregnancy rate in frozen-thawed embryo transfer cycles is usually lower than that of fresh transferred embryos (11-12). In addition, the chance of live birth following FET is further reduced by the increased incidences of pregnancy damage (13). 

In previous study by Salumets *et al *the rate of biochemical pregnancy and clinical abortion was reported 15-20% and 20-25% after FET, respectively (14). Although, the reasons for impaired pregnancy and elevated spontaneous abortion rates after FET are not completely understood, they are most likely caused by the damage to embryos occurring during the freezing and thawing procedures (15-16). The pregnancy outcomes after the FET is known to be dependent on multiple clinical factors, including the age of the woman, method of oocyte fertilization (i.e.In Vitro Fertilization (IVF or intra-cytoplasmic sperm injection (ICSI)), duration of infertility, FSH serum level, reasons for embryo cryopreservation, type of infertility (primary or secondary), and endometrial thickness on the day of embryo transfer (3,17). Considering the importance of FET in ART, this study aimed to evaluate the clinical factors that may influence the pregnancy rate after FET, and to provide more precise outcome advice for couples. 

## Materials and methods

Permission to perform this study was given by the ethics committee of Research and Clinical Center for Infertility, Shahid Sadoughi University of Medical Sciences, Yazd, Iran. In this retrospective cross sectional study, we reviewed the clinical records of 347 women who were received FET treatment registered over a 2-year period from April 2009-2011 at the Research and Clinical Center for Infertility, Shahid Sadoughi University of Medical Sciences, Yazd, Iran.

The subjects with a history of hysteroscopy/ curettage, endocrine disorders (i.e. diabetes and hypothyroidism) were excluded from the study. The cycles were also excluded if the embryos were derived from donated gametes, or embryo transfer performed in natural cycles. This study includes the cycle on which endometrial preparation was performed by hormones administration. The clinical factors data were collected consisting of maternal and paternal age at the time of embryo transfer, duration of infertility, FSH serum level, type of assisted reproduction employed (IVF or ICSI procedure), reasons for embryo cryopreservation, endometrial thickness on the day of embryo transfer, and duration of FET treatment. The duration of treatment defined as from the day of embryo storage to the day of FET.

All embryos were cryopreserved by vitrification method. Controlled ovarian stimulation was achieved mainly using the gonadotropin-releasing hormone agonist (GnRH) for pituitary suppression and recombinant FSH in a long protocol. The subjects undergo pituitary desensitization by a long acting GnRh analogue administered in the luteal phase of the previous cycle. All these then receive exogenous estrogen pills (6 mg estradiol valerate/day, Aburaihan Co., Tehran, Iran) therapy for endometrial preparation before the embryo transfer. Endometrial thickness was documented by transvaginal ultrasonography. When endometrial thickness had reached 8mm or more, progesterone (100 mg, IM, Iran hormone, Iran) 400 mg twice daily were commenced.

Embryos were thawed two days before progesterone administration. Embryos were accepted for transfer if they retained ≥50% of blastomeres intact after thawing. Embryos were transferred using a Labotect catheter (Labotect, Gottingen Germany). Confirmation of a successful implantation was performed by detecting an increased serum human chorionic gonadotrophin (β-HCG) concentration (>50 IU/ml) 2 days post-frozen embryo transfer, and was considered as positive biochemical pregnancy. Clinical pregnancy was defined by a presence of a gestational sac and fetal heart activity by ultrasound at 7 weeks of pregnancy.

**Statistical analysis**

Statistical analyses were performed using the SPSS statistical package, version 15.0 (SPSS Inc., Chicago. IL, USA) between-group differences. Of normally distributed continuous variables were assessed by Student’s t test. Significant differences were evaluated by the Chi-square test to compare the non-continuous variables. The data were expressed as mean±SD. P-value<0.05 was considered statistically significant.

## Results

The present study was conducted on 372 patients who received FET. Patients’ characteristics are summarized in table I. The data concerning the pregnancy rate based on etiology of infertility presented in table II. Table III showed the clinical factors influencing clinical pregnancy rates. As noted, the pregnancy rate after FET in women aged <35 was 57.7%; In contrast, the pregnancy rate in patients aged>35 was 29.2%. Statistical analysis showed that young (<35) and old (35-40) mothers have a significant differences in pregnancy rate following FET (p<0.0001). In our study, pregnancy rate was 54.4%. In two cases, cryopreservation was done for endometrial insufficiency which their pregnancy rate was 50%. 

Pregnancy rate in agonist protocol subjects (106 cases) were 52.5% that showed an insignificant difference to antagonist protocol patients (88 cases) who had 51.8% positive clinical pregnancy. Concerning to the type of treatment, our results indicated a slightly difference between the IVF subjects (80 cases) with pregnancy rate 52.3%, when compared to ICSI patients (114 cases) with 52.1% of pregnancy rate (p=1). Pregnancy rate in women, who had the FSH serum level more than 10 IU/ml, showed a significant difference in comparison to the subjects with the FSH serum level less than 10 IU/ml (0.0001). Our data showed that the some clinical factor such as endometrial thickness and duration of treatment had no influence on pregnancy outcomes (p=0.916). The detailed data are presented in table III.

**Table I:** Characteristics of patients studied

**Variable**	**mean±SE**
Female age (year)	30.22 ± 4.83
Male age (year)	39.4 ± 5.42
Infertility duration (year)	8.26 ± 4.41
Basal FSH (IU/ml)	6.8 ± 3.02
Embryos transferred	2.45 ± 0.75

Parameters expressed as mean±SE

**Table II:** The frequency distribution of pregnancy rate based on etiology of infertility in patients studied

**Etiology of infertility**	**Number of patients**	**Pregnancy rate (%)**
Male factor	68	48.9
Poly cystic ovary	68	53.54
Tubal factor	8	47.1
Endometriosis	5	62.5
Hypothalamic amenorrhea	1	33.3
Unexplained	24	54.5
Mixed	20	5.37
Total	194	52.2

Parameters expressed as mean±SD or percentage as appropriate

**Table III:** Pregnancy rate based on clinical factors in patients studied

**Clinical factors **		**Number of patients**	**Clinical pregnancy rate (%)**	**p-value**
Female age				
	<35	173	57.7	0.0001
	>35	21	29.2	
Reason for embryo cryopreservation				
	Preserve the excess embryos	101	50.2	0.717
	Preventing ovarian hyperstimulation	91	54.5	
	Endometrial insufficiency	2	50	
Primary ovarian hyperstimulation protocol				
	Agonist	106	52.5	0.487
	Antagonist	88	51.8	
ART techniques				
	IVF	80	52.3	1.00
	ICSI	114	52.1	
Type of infertility				
	Primary	168	52	1.00
	Secondary	26	53.1	
FSH level				
	>10 IU/ml	187	56.3	0.0001
	≤10 IU/ml	7	17.5	
Endometrial thickness				
	<9 mm	137	50.4	0.916
	≥ 9 mm	57	57	
Duration of treatment				
	<16 days	118	52.4	0.916
	≥16 days	76	51.7	

Parameters expressed as mean±SD or percentage as appropriate.

Chi-square and Student’s *t *test were used.

## Discussion

The present retrospective study was carried out to provide a better understanding of the clinical factors in predicting the pregnancy outcome of frozen-thawed embryo transfers using data from frozen embryo transfer cycles performed at the Research and Clinical Center for Infertility, Shahid Sadoughi University of Medical Sciences, Yazd, Iran. Based on our results, women age and FSH serum level are the most important clinical factors influencing pregnancy rate after frozen embryo transfer (Table III). The women age is the most important factor predicting success with fresh or FET (14, 23, 31-35). It was not concisely determined whether these findings could be related to lower quality of embryo or whether other factors were caused these results (14, 23). 

In agreement with the previous reports, in our study, women age showed as critical factor in pregnancy rate following FET (14, 23, 31-35). Because of presented limitations and no complete recorded data, we were not able to evaluate the scoring embryo at cryopreservation time. Our results confirm the reported negative association between the maternal age and pregnancy outcomes in FET technique (31-35). In previous reports, a reduced pregnancy rate following FET, as well as, following the transfer of fresh embryos was recorded with increasing maternal age (31-35).

According to the study by Ashrafi *et al* the women age did not affect pregnancy rate in FET protocol (22). They concluded that the quality of embryos was crucial factor determining the success of FET. Other studies also revealed the FSH level caused a remarkable effect on pregnancy rate in FET (19, 22-24, 32). In another investigation, Kassab *et al* reported inverse correlation between basal serum FSH levels before fresh IVF/ICSI cycle with pregnancy outcome in FET cycles (23). The other factors consisting reason for embryo cryopreservation, ovulation-stimulating protocol, type of infertility, endometrial thickness at embryo transfer day, and the duration of treatment, have not influence on the pregnancy rate after FET (Table III). 

The results of El-Toukhy *et al* showed an endometrial thickness of 9-14 mm measured on the day of progesterone supplementation was associated with higher implantation and pregnancy rates compared with an endometrial thickness of 7-8 mm (25). It seems that endometrial thickness and appropriate endometrial quality in FET cycles are more important than duration of endometrial preparation to get good results in implantation and pregnancy rates (19, 25, 27-30). In the present study, we found no correlation between the duration and method of endometrial preparation with the pregnancy rate (Table III). Although, there is evidence indicating a higher pregnancy rate in GnRH agonist compared with the GnRH antagonist treatments to support luteal phase (36). Some reports indicated that the potential for frozen embryos to implant and develop following transfer is independent of the GnRH-antagonist/ GnRH-agonist protocols (37). This study results are consistent with the present result concerning the influence of type of ovarian hyperstimulation on pregnancy rate in FET procedure. 

In agreement with the present study, Rimm *et al* reported the insignificant differences between ART (IVF and ICSI) protocols with the pregnancy outcomes (38). The other investigations also were showed a lower chance of successful conception in ICSI protocol (39). The ART techniques are expensive; require a considerable commitment of time and energy for infertile couples. Therefore, finding out of the affecting factor is a great way to improve their chances of conceiving. The other factors affect pregnancy rate and their implications for ART outcome need to be further investigated.

## Conclusion

In summary, women age and FSH serum levels are the crucial factors to get success in ART and FET techniques, and should be considered. The other clinical factors (i.e. reason for embryo cryopreservation, ovulation-stimulating protocol, type of infertility, endometrial thickness at embryo transfer day, and the duration of treatment) seem to have no effects on the pregnancy outcome following FET.

## Footenote

***This article extracted from fellowship course thesis. (Elham Rahmani)***
